# Tumor Genotyping and Homologous Recombination Repair Gene Variants in Patients With Epithelial Ovarian Cancer: Is Pathogenic Enough?

**DOI:** 10.3389/fonc.2021.683057

**Published:** 2021-06-01

**Authors:** Elena Fountzilas, Vassiliki Kotoula, Georgia-Angeliki Koliou, Michalis Liontos, Kyriaki Papadopoulou, Eleni Giannoulatou, Alexios Papanikolaou, Ioannis Tikas, Sofia Chrisafi, Davide Mauri, Kyriakos Chatzopoulos, Florentia Fostira, Dimitrios Pectasides, Georgios Oikonomopoulos, Dimitra Aivazi, Angeliki Andrikopoulou, Anastasios Visvikis, Gerasimos Aravantinos, Flora Zagouri, George Fountzilas

**Affiliations:** ^1^ Second Department of Medical Oncology, Euromedica General Clinic of Thessaloniki, Thessaloniki, Greece; ^2^ Department of Pathology, Aristotle University of Thessaloniki, School of Health Sciences, Faculty of Medicine, Thessaloniki, Greece; ^3^ Laboratory of Molecular Oncology, Hellenic Foundation for Cancer Research/Aristotle University of Thessaloniki, Thessaloniki, Greece; ^4^ Section of Biostatistics, Hellenic Cooperative Oncology Group, Data Office, Athens, Greece; ^5^ Department of Clinical Therapeutics, Alexandra Hospital, National and Kapodistrian University of Athens School of Medicine, Athens, Greece; ^6^ Computational Genomics Laboratory, Victor Chang Cardiac Research Institute, Darlinghurst, NSW, Australia; ^7^ The University of New South Wales, Kensington, NSW, Australia; ^8^ First Department of Obstetrics and Gynecology, Papageorgiou Hospital, Aristotle University of Thessaloniki, School of Health Sciences, Faculty of Medicine, Thessaloniki, Greece; ^9^ Department of Medical Oncology, Medical School, University of Ioannina, Ioannina, Greece; ^10^ Society for Study of Clonal Heterogeneity of Neoplasia (EMEKEN), Ioannina, Greece; ^11^ Molecular Diagnostics Laboratory, InRASTES, National Centre for Scientific Research Demokritos, Athens, Greece; ^12^ Oncology Section, Second Department of Internal Medicine, Hippokration Hospital, Athens, Greece; ^13^ Second Department of Medical Oncology, Metropolitan Hospital, Piraeus, Greece; ^14^ Third Department of Medical Oncology, Agii Anargiri Cancer Hospital, Athens, Greece; ^15^ Second Department of Medical Oncology, Agii Anargiri Cancer Hospital, Athens, Greece; ^16^ Aristotle University of Thessaloniki, Thessaloniki, Greece; ^17^ Department of Medical Oncology, German Oncology Center, Limassol, Cyprus

**Keywords:** biomarker, BRCA, co-mutation, homologous recombination repair, predictive, prognostic

## Abstract

Our hypothesis was that the predictive accuracy of pathogenic variants in genes participating in the homologous recombination repair (HRR) system in patients with epithelial ovarian cancer (EOC) could be improved by considering additional next-generation sequencing (NGS) metrics. NGS genotyping was performed in tumor tissue, retrospectively and prospectively collected from patients with EOC, diagnosed from 8/1998 to 10/2016. Variants were considered clonal when variant allele frequencies corresponded to >25%. The primary endpoint was overall survival (OS). This study included 501 patients with EOC, predominantly with high-grade serous (75.2%) and advanced stage tumors (81.7%); median age was 58 years (22-84). Pathogenic and clonal pathogenic variants in HRR and/or TP53 genes were identified in 72.8% and 66.5% tumors, respectively. With a median follow-up of 123.9 months, the presence of either pathogenic or clonal pathogenic HRR-only variants was associated with longer OS compared to HRR/*TP53* co-mutation (HR=0.54; 95% CI, 0.34-0.87, Wald’s p=0.012 and HR=0.45; 95% CI, 0.27-0.78, Wald’s p=0.004, respectively). However, only the presence of clonal HRR-only variants was independently associated with improved OS (HR=0.55; 95% CI, 0.32-0.94, p=0.030). Variant clonality and co-occuring TP53 variants affect the predictive value of HRR pathogenic variants for platinum agents in patients with EOC.

**Clinical Trial Registration:**

[ClinicalTrials.gov], identifier [NCT04716374].

## Introduction

Precision oncology uses tumor histopathology, genomic/molecular alterations and immune profile, in combination with patient’s clinical characteristics and comorbidities to select the optimal treatment (1). Ovarian cancer is one of the characteristic clinical settings, where precision medicine has led to a significant improvement in patient outcomes. Specifically, tumor and germline testing provide clinically relevant information for the use of innovative treatments, including poly ADP-ribose polymerase (PARP) inhibitors and immunotherapy (1–4). On this basis, the American Society of Clinical Oncology (5), the National Cancer Comprehensive Network (6), the Society of Gynecologic Oncology (7) and the European Society of Medical Oncology (8) recommend the implementation of tumor molecular profiling at the time of diagnosis of epithelial ovarian cancer (EOC).

These recommendations focus on the identification of pathogenic tumor and/or germline variants (mutations) in BRCA1/2 and other genes participating in the homologous recombination repair (HRR) of double-strand DNA breaks. When these genes are non-functional, cells develop homologous recombination repair deficiency (HRD) and are rendered sensitive to platinum-based chemotherapy (9, 10) and PARP inhibition (11). The first approvals of PARP inhibitors in patients with recurrent disease (12, 13) were based on pathogenic BRCA1/2 mutations. In recurrent platinum sensitive disease, initial response to platinum treatment was the most consistent parameter associated with response to PARP inhibitors (14–16). Based on these data, PARP inhibitors have been approved as maintenance treatment of adult patients with recurrent epithelial ovarian, fallopian tube, or primary peritoneal cancer, who are in a complete or partial response to platinum-based chemotherapy (17, 18). Thus, the clinical phenotype, i.e., platinum sensitivity, appears to be a surrogate for response to PARP inhibitors in EOC, in the absence of reliable HRD testing (19–21). As these drugs are now being used as front-line treatment (21), and since more than 70% of patients does not respond to therapy or progresses soon after standard platinum-based chemotherapy or during maintenance therapy with PARP inhibitors (20), it is critical to improve currently used markers predictive of platinum sensitivity.

Tumor molecular profiling, which is performed in routinely processed formalin-fixed paraffin-embedded (FFPE) tissues, is recommended for newly diagnosed EOC. The presence of a pathogenic BRCA1/2 or any HRR gene mutation in a tumor is not synonymous and interchangeable with HRD, and it does not necessarily signify a non-functional gene status (22, 23). Guidelines for reporting and interpreting the clinical relevance of e.g., BRCA1/2 variants in tumors usually focus on the accurate annotation of variant pathogenicity and on increasing the sensitivity of variant detection, by taking into account the variant load (24, 25), or without such consideration (26). However, a higher rate of a pathogenic allele in a tumor would indicate the presence of a clonal alteration driving tumor evolution (27) and in the case of HRR genes, loss of function. Additionally, the clinical relevance of HRR co-mutations has seldom been addressed in EOC (28), even though this phenomenon is common in this context (29).

Here, we hypothesized that the mere presence of pathogenic variants in genes participating in the HRR system cannot sufficiently predict benefit from platinum-based chemotherapy and improved patient outcomes. In this context, we retrospectively examined the mutational profile of EOC and assessed additional variant parameters that are obtained with next-generation sequencing (NGS), including load of pathogenic HRR variant in the examined samples, and concurrent pathogenic variants in HRR genes and TP53 to assess whether these parameters could provide more reliable information on predicting response to platinum agents compared to HRR pathogenic variants alone.

## Materials and Methods

### Patients

Patients with epithelial ovarian adenocarcinoma with archival tumor tissue available for analysis were identified through the Hellenic Cooperative Oncology Group (HeCOG)’s tumor repository. Patients had been diagnosed from 8/1998 to 10/2016 and had received treatment at HeCOG-affiliated institutions following standard international guidelines. Patient demographics, tumor histopathological characteristics, treatment regimens and outcome data were recorded from the HeCOG electronic clinical database.

### Samples and Genotyping

Tumor tissue processing and all NGS genotyping were performed at the Laboratory of Molecular Oncology (Hellenic Foundation for Cancer Research/AUTH). Paraffin H&E sections from the retrieved tissue blocks were centrally reviewed for tumor histology and tissue adequacy for DNA extraction and were marked for macrodissection along with tumor DNA content [(former tumor cell content (TCC%)] assessment (30, 31). DNA was extracted from macrodissected tissue fragments with the QIAamp^®^ DNA mini kit (Qiagen, Hilden, Germany), measured in a Qubit fluorometer (Thermo Fisher Scientific, Paisley, UK), and genotyped with NGS in an Ion Torrent Proton sequencer (Thermo Fisher Scientific) by using a previously published custom panel (32). Following stringent variant quality filtering (30), 500 tumors were considered for analysis, with median TCC 68.3% (interquartile range [IQR] 53.3% – 80%), average mean depth at 3854 (IQR 2166.5 – 5379.5) and average uniformity of 86.03% (IQR 81.15% – 89.59%). The same panel was applied for germline DNA genotyping in patients with available peripheral blood samples, yielding 247 technically informative samples.

### Variant Classification

The ~59000 informative variants were annotated with ANNOVAR (33) v. March 2019. Amino acid and splice site changing variants with minor allele frequencies <0.1% in the European population were considered as mutations. Of these, pathogenic variants were called based on COSMIC, CLINSIG and fathmm scores. The present analysis was restricted in HRR (ATM, BRCA1, BRCA2, CHEK2, FANCD2, MRE11A, PALB2, RAD50) and TP53 gene variants. Pathogenic variants were further classified as clonal for stringently obtained variant allele frequencies (VAFs) corresponding to >25% (34), and position-loss of heterozygocity (LOH) for VAFs corresponding to >65% (35, 36) of 0.5XTCC% (30). The subset of clonal pathogenic variants included position-LOH and that of pathogenic variants included both clonal and position-LOH, with a respectively aggravating impact in terms of gene deactivation. Although this is only an approximate estimation of the variant load in an FFPE tumor sample, it may still be informative for the assessment of clinical samples on a routine basis (31, 35, 36).

Based on the panel targets (32), HRR pathogenic variants might have been missed in both tumors and blood samples. Therefore, in patients with informative blood samples, germline status was considered for those who tested positive with the custom panel in matched blood/tumor samples, as well as for those who had genetic test results with a multigene panel (37). In cases without matched blood samples, known cancer predisposing variants in the targeted genes with sample VAFs >65% independently of TCC% were considered as suspected germline variants.

### Statistical Analysis

Patient, tumor characteristics and mutation classes were summarized using descriptive statistics, including counts with the corresponding percentages (for categorical variables) and medians with ranges (for continuous variables). The chi-square test was used for comparisons of categorical data and the Kruskal-Wallis or the Wilcoxon rank-sum test for comparisons between categorical and continuous variables. Overall survival (OS) was defined as the time from ovarian cancer diagnosis to the date of death from any cause. Alive patients were censored at the last follow-up date. Progression free survival (PFS) was defined as the time from initiation of first-line chemotherapy to the first documented progression, death from any cause or last contact, whichever occurred first and was estimated only among patients treated with first-line chemotherapy. Time-to-event endpoints were assessed in the entire cohort upon exclusion of patients with mucinous tumors and separately among patients with high grade serous tumors of advanced stage (III or IV), using the Kaplan-Meier product limit method. The complementary log-log transformation was used to calculate the 95% confidence intervals (CI) for the median values and the log-rank test was performed for comparison of survival distributions. Our analysis focused on patients with HRR and/or TP53 pathogenic variants. The effect of clinicopathological parameters of interest (age, stage, histology, performance status (PS), family history of cancer) and of the presence of (clonal) pathogenic variants in HRR/TP53 genes on OS and PFS was estimated by univariate Cox regression models. Departures from the proportional hazards assumption were assessed using time dependent covariates. The group of patients with (clonal) pathogenic variants in both HRR and TP53 genes was used as the reference group and was compared to the group of patients with a) HRR-only and b) TP53-only variants. Because of the aforementioned selective targeting by the applied panel, our analysis was limited to the subgroup of patients whose tumors harbored variants in these genes. Multivariate models adjusting for age, stage (I-II, III-IV), PS (0, 1-3) and histology (high grade serous vs. other) were applied to estimate the independent effect of (clonal) pathogenic variants on patients’ outcomes. In the subpopulation of patients with high grade serous tumors of advanced stage, the effect of (clonal) pathogenic variants was adjusted for age and PS only. All tests were two-sided at a 5% level of significance. Analysis was performed using the SAS (version 9.3, SAS Institute Inc., Cary, NC) software.

## Results

### Patient Clinicopathological Characteristics

In total, 501 patients with ovarian adenocarcinoma were included in the study. Median age at ovarian cancer diagnosis was 58 years (range 22-84). Tumor histological types included predominantly high-grade serous (n=377 patients, 75.2%), followed by endometrioid (n=58, 11.6%) and clear cell carcinomas (n=30, 6%). All except 3 patients (of 488 patients with available data) underwent surgery at initial diagnosis, most commonly total abdominal hysterectomy with bilateral salpingo-oophorectomy (392 patients, 82%). Residual disease <2cm was reported in 71% (218 of 307 with available data) patients. Patient clinicopathological characteristics are summarized in **Table 1**.

**Table 1:** Patient and tumor characteristics.

	Total (N=501)
**Age (N=499)**	57.7(21.7,83.9)
**Previous Other Cancer (N=478)**	
No	466(97.5)
Yes	12(2.5)
**Family Other Cancer (N=463)**	
No	300(64.8)
Yes	163(35.2)
**Initial stage (N=496)**	
I	58(11.7)
II	33(6.7)
III	350(70.6)
IV	55(11.1)
**PS (N=478)**	
0	357(74.7)
1	92(19.2)
2	26(5.4)
3	3(0.63)
**Histology (N=501)**	
HGSOC	377(75.2)
LGSOC	11(2.2)
Clear cell	30(6.0)
Endometrioid	58(11.6)
Mucinous	25(5.0)
**Surgery (N=488)**	
No	3(0.6)
Yes	485(99.4)
Type of surgery (N=478)*	
BSO	18(3.8)
TAH & BSO	392(82.0)
TAH & USO	35(7.3)
USO	5(1.0)
Other	28(5.9)
Residual disease (N=307)*	
0 cm	143(46.6)
<2 cm	75(24.4)
2-5 cm	48(15.6)
>5 cm	41(13.4)
**Chemotherapy (N=488)**	
No	2(0.41)
Yes	486(99.6)
**Type of treatment (N=486)**	
Adjuvant	13(2.7)
Front-line	473(97.3)

Values presented as Median (min, max) or N (column %).

*Percentages for the type of surgery and residual disease were calculated out of the total number of patients with available data that had undergone surgery.

BSO, bilateral salpingo-oophorectomy; HGSOC, high-grade serous ovarian cancer; LGSOC, low-grade serous ovarian cancer; N, number; TAH, total abdominal hysterectomy; USO, unilateral salpingo-oophorectomy.

### Tumor Molecular Profiling

**Figure 1:**
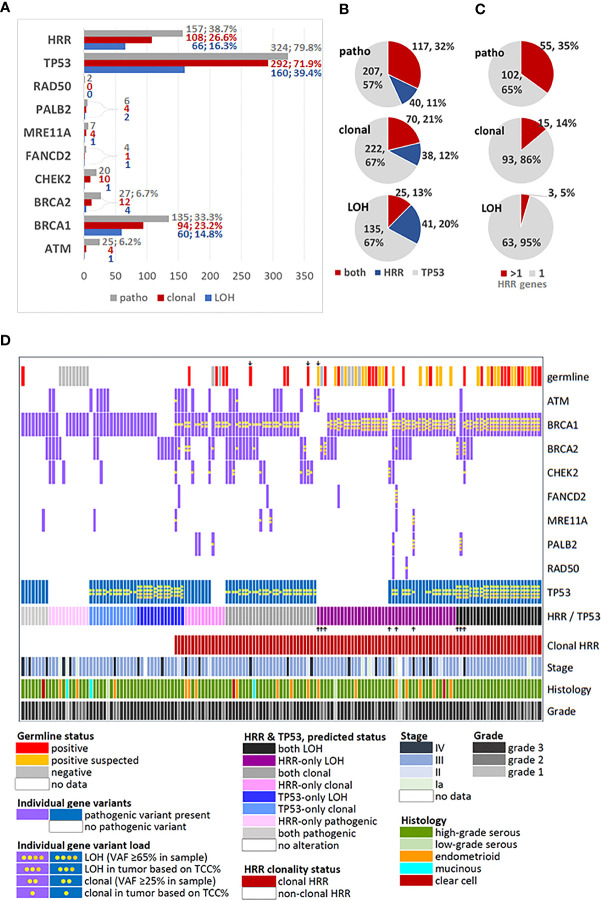
Description and distribution of alterations in HRR and TP53 genes. **(A)** Incidence of alterations for each studied gene and for grouped HRR genes. Variants were classified as pathogenic, by simple presence, and as clonal or position-LOH, based on mutation and tumor DNA load in the examined sample. X-axis: number of affected tumors. Percentages are shown for rates >5% among all tumors with pathogenic variants (n=406). **(B)** Distribution of TP53/HRR gene co-mutations in the same tumor, among tumors bearing the same class of alteration in any of these genes. **(C)** Distribution of multiple HRR gene pathogenic variants in the same tumor, among tumors bearing the same class of alteration in these genes. **(D)** Map showing profiled HRR and TP53 gene mutations among tumors bearing any class of alterations in these genes, in comparison to germline mutation status and standard clinicopathological parameters. Up or down showing arrows: non-BRCA1 alterations. Tumors with only TP53 mutations and non-mutated tumors were not included in this chart.

Among 500 informative tumors, pathogenic variants in any gene of the panel were identified in 406 (81.2%); of these, 364 (89.7%) had pathogenic variants in HRR and/or TP53 genes (**Figure 1A**). The most frequently affected genes were TP53 in 324 (of 500, 64.8%) and BRCA1 in 135 (27%) tumors, while pathogenic variants in all examined HRR genes were observed in 157 (31.4%) tumors. Clonal variants in HRR and/or TP53 genes were identified in 330/496 (66.5%) tumors. Clonal TP53, BRCA1 and HRR gene variants were observed in 292 (58.4%), 94 (18.8%) and 108 (21.6%) tumors, respectively. Position-LOH was observed in 201/495 (40.6%) tumors. TP53 was affected in 160 (of 500 informative tumors, 32%), BRCA1 in 60 (of 498 informative, 12.0%), and HRR genes in 66 (of 495 informative, 13.3%) tumors.

The majority of tumors had one pathogenic variant, while 174 (34.8%) tumors had ≥2 variants. Simultaneous presence of pathogenic HRR and TP53 variants was observed in 117 of 364 (32.1%) tumors with alterations in these genes. The simultaneous presence of the same alteration class in TP53 and HRR genes declined from pathogenic to clonal to position-LOH (**Figure 1B**). Similarly, among the 157 tumors bearing alterations in HRR genes, multiple pathogenic, clonal and position-LOH alterations in the same or in different genes were observed with respectively declining incidence (**Figure 1C**).

The profiles of HRR gene alteration classes in the 157 affected tumors are shown in **Figure 1D**. Among these tumors, the majority (117/157, 74.5%) had simultaneous pathogenic TP53 alterations. In addition, alterations in ≥2 HRR genes were observed in 62/157 (39.5%) of all HRR affected tumors and in 45/117 (38.5%) of HRR/TP53 co-mutated tumors. Only 19/157 (12.1%) HRR affected tumors had non-BRCA1 pathogenic variants in single HRR genes (10 BRCA2, 5 CHEK2, 3 ATM and 1 BLM), precluding separate statistical analysis on single HRR genes co-mutated with TP53. This map also shows the strong association between clonal pathogenic HRR variants and positive germline status, validated (chi-square p<0.0001) and validated/suspected (Fisher’s exact p<0.0001). However, these results cannot be generalized because these only pertain to the subgroup of 86 patients with known germline status (17.2% of the cohort).

Regarding all tumors informative for HRR and TP53, pathogenic HRR/TP53 variants were observed in 117 (32.1%) of 364 tumors, while clonal HRR/TP53 in 70 (21.2%) of 330 tumors.

In the entire cohort, the presence of pathogenic variants in both HRR and TP53 genes was associated with advanced stage disease (p=0.024). Patients carrying tumors with clonal TP53-only pathogenic variants were of older age as compared to those with HRR-only clonal pathogenic variants (median age: 59.3 vs. 52.2, Wilcoxon rank-sum p<0.001) or clonal pathogenic co- variants in HRR/TP53 genes (median age: 59.3 vs. 54.9, p=0.020). Tumors with position LOH and clonal variants were more frequent in high-grade serous ovarian cancer (HGSOC) (both p-values <0.001) (**Supplementary Table 1**).

### Clinical Outcomes

#### All Patients

Survival analysis was performed after excluding patients with mucinous tumors. At the time of analysis, with a median follow-up of 123.9 months (95% CI 115.9-132.1), 308 deaths had occurred. Among 474 patients with available data, the median OS was 66.8 months (95% CI 58.2-75.2). Increasing age, higher stage (stage III-IV vs. I-II), histology (HGSOC vs. other) and performance status (1-3 vs. 0) were associated with shorter OS univariately (**Supplementary Table 2**).

#### Patients With HRR and/or TP53 Mutated Tumors, Excluding Mucinous

**Figure 2:**
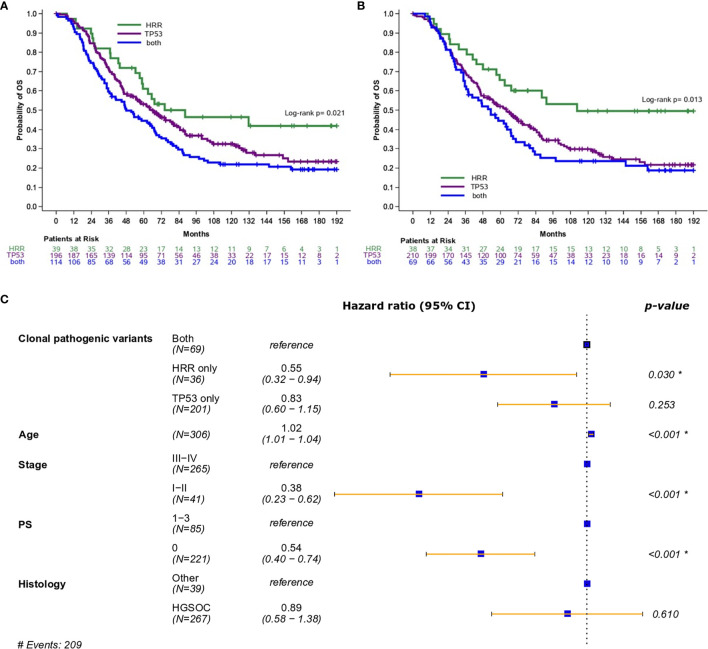
**(A)** Overall survival based on the presence of HRR and/or TP53 pathogenic variants (excluding mucinous tumors). **(B)** Overall survival based on the presence of HRR and/or TP53 clonal pathogenic variants (excluding mucinous tumors). **(C)** Forest plot of hazards ratios showing the risk of death for patients with HRR and/or TP53 clonal pathogenic variants upon adjustment for clinicopathological parameters (excluding mucinous tumors). *Statistically significant parameters.

Importantly, the presence of both pathogenic and clonal HRR-only variants was associated with longer OS compared to HRR/*TP53* co-mutation (HR=0.54; 95% CI, 0.34-0.87, Wald’s p=0.012, **Figure 2A**, and HR=0.45; 95% CI, 0.27-0.78, Wald’s p=0.004, **Figure 2B**, respectively).

In multivariate analysis, the presence of pathogenic variants did not retain its favorable prognostic significance for OS (HR=0.69; 95% CI, 0.43-1.12, p=0.14). On the contrary, the presence of clonal HRR-only variants was independently associated with improved OS (HR=0.55; 95% CI, 0.32-0.94, p=0.030) (**Figure 2C**).

PFS analysis was also performed in patients without mucinous tumors who received first-line platinum-based treatment. Among 452 patients, 339 progressed (75%) at a median PFS of 24.9 months (95% CI 22.1-31.5). Both the presence of pathogenic and clonal HRR-only variants compared to HRR/*TP53* co-mutations was univariately associated with longer PFS (HR=0.56; 95% CI, 0.36-0.88, p=0.012 and HR=0.56; 95% CI, 0.34-0.93, p=0.023, respectively), but did not remain independently significant in multivariate analysis (details in **Table 2**).

**Table 2:** Cox regression analysis with respect to OS and PFS in patients with HRR and/or *TP53* pathogenic variants.

	Univariate				Multivariate*				
		Event/Total	HR (95% CI)	p-value	Event/Total		HR (95% CI)	p-value	
**Entire cohort with HRR and/or TP53 pathogenic variants-excluding mucinous tumors**	***OS***								
	**Pathogenic variants**			**0.023**				0.23	
	HRR only	21/39	0.54 (0.34-0.87)	**0.012**	21/39		0.69 (0.43-1.12)	0.14	
	TP53 only	128/196	0.77 (0.59-1.01)	0.058	125/188		0.83 (0.63-1.10)	0.19	
	Both	89/114	Reference	–	88/111		Reference	–	
	**Clonal pathogenic variants**			**0.016**				0.091	
	HRR only	18/38	0.45 (0.27-0.78)	**0.004**	18/36		0.55 (0.32-0.94)	**0.030**	
	TP53 only	142/210	0.84 (0.61-1.15)	0.27	138/201		0.83 (0.60-1.15)	0.25	
	Both	53/69	Reference	–	53/69		Reference	–	
	PFS**								
	**Pathogenic variants**			**0.036**				0.19	
	HRR only	24/39	0.56 (0.36-0.88)	**0.012**	24/39		0.67 (0.42-1.05)	0.081	
	TP53 only	145/186	0.83 (0.64-1.08)	0.16	140/179		0.86 (0.66-1.12)	0.26	
	both	92/112	Reference	–	91/110		Reference	–	
	**Clonal pathogenic variants**			0.055				0.16	
	HRR only	22/37	0.56 (0.34-0.93)	**0.023**	22/36		0.62 (0.38-1.03)	0.063	
	TP53 only	160/201	0.95 (0.70-1.30)	0.75	154/193		0.93 (0.68-1.27)	0.63	
	Both	54/68	Reference	–	54/68		Reference	–	
**Advanced HGSOC**	***OS***								
	**Pathogenic variants**			0.10					0.31
	HRR only	19/29	0.61 (0.37-1.02)	0.057	19/29	0.75 (0.45-1.26)			0.28
	TP53 only	102/146	0.79 (0.58-1.06)	0.12	101/141	0.81 (0.59-1.09)			0.17
	Both	74/91	Reference	–	73/90	Reference			
	**Clonal pathogenic variants**			**0.023**				**0.044**	
	HRR only	13/26	0.42 (0.23-0.78)	**0.006**	13/26		0.47 (0.25-0.87)	**0.016**	
	TP53 only	116/161	0.84 (0.59-1.19)	0.32	114/155		0.75 (0.53-1.07)	0.11	
	Both	44/55	Reference	–	44/55		Reference	–	
	PFS**								
	**Pathogenic variants**			0.23	–				
	HRR only	22/29	0.69 (0.43-1.10)	0.12					
	TP53 only	114/142	0.83 (0.62-1.11)	0.21					
	Both	76/90	Reference	–					
	**Clonal pathogenic variants**			0.088				0.17	
	HRR only	17/26	0.55 (0.31-0.96)	**0.037**	17/26		0.58 (0.33-1.02)	0.058	
	TP53 only	129/157	0.94 (0.67-1.33)	0.74	125/151		0.85 (0.60-1.21)	0.37	
	Both	44/54	Reference	–	44/54		Reference	–	

HR, hazard ratio; CI, confidence interval; HRR, homologous recombination repair; HGSOC, high-grade serous ovarian cancer; PFS, progression-free survival; OS, overall survival.

*Adjusting for age and performance status.

**Assessed in patients treated with 1^st^ line chemotherapy.

Statistically significant p-values are shown in bold.

Even though the primary analysis focused on patients with pathogenic variants in HRR and/or TP53 genes, we further evaluated our results in the entire cohort including patients carrying tumors without any pathogenic variants or with pathogenic variants in other genes. The replication of the analysis in the entire cohort yielded results consistent with the ones previously obtained, thus ensuring robustness of our analysis (**Supplementary Figure 1**).

#### Patients With Advanced HGSOC

**Figure 3:**
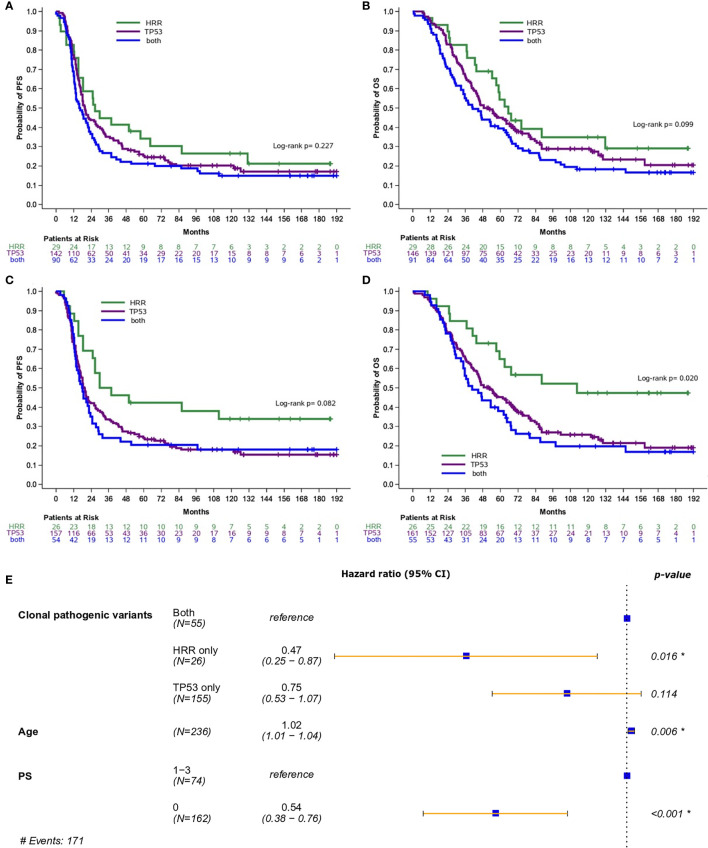
Analysis was performed in patients with high-grade serous ovarian cancer. **(A)** Progression-free survival (PFS) and **(B)** Overall survival (OS) based on the presence of pathogenic variants in HRR and/or TP53 genes. **(C)** PFS and **(D)** OS based on the presence of clonal pathogenic variants in HRR and/or TP53 genes. **(E)** Forest plot of hazard ratios showing the risk of death for patients with HRR and/or TP53 clonal pathogenic variants upon adjustment for clinicopathological parameters. *Statistically significant parameters.

Additionally, the prognostic role of pathogenic and clonal pathogenic variants was explored in patients with advanced HGSOC. The presence of pathogenic variants in HRR and/or TP53 genes did not appear to be associated with PFS (p=0.23, **Figure 3A**) or OS (p=0.10, **Figure 3B**) in this subgroup of patients. By contrast, the presence of clonal HRR-only mutations compared to HRR/*TP53* co-mutations was associated with longer PFS (HR=0.55; 95% CI, 0.31-0.96, p=0.037, **Figure 3C**) and OS (HR=0.42, 95% CI, 0.23-0.78, p=0.006, **Figure 3D**). Finally, clonal HRR-only variants were independently associated with longer OS compared to *HRR/TP53* co-mutations (HR=0.47, 95% CI, 0.25-0.87, p=0.016) (**Figure 3E**). Detailed data on univariate and multivariate analysis in patients with advanced HGSOC is shown in **Table 2**.

## Discussion

Molecular alterations in HRR genes have been associated with clinical benefit from chemotherapy and/or PARP inhibitors in patients with EOC. Therefore, the performance of tumor molecular profiling is currently recommended by international guidelines (6–8) at initial diagnosis, among other reasons, for the modification of the treatment plan. We show that tumor molecular profiling reveals additional parameters that can improve the predictive and prognostic role of the mere presence of HRR gene mutations. In our study, patients with clonal variants in HRR genes without concurrent *TP53* pathogenic variants had improved PFS and OS compared to patients with HRR/*TP53* co-mutated tumors. Similar findings were observed in advanced stage HGSOC, the predominant histological type of clinical interest.

Our initial hypothesis was that the predictive adequacy of pathogenic variants might be improved by taking into account additional variant metrics that are provided with each tumor NGS genotyping test. Indeed, in our study the presence of pathogenic variants in HRR genes was not independently associated with OS or PFS, either in the total population or in patients with advanced stage HGSOC. When taking into consideration the clonality of the respective variants, we observed a significant improvement of their prognostic and predictive value. In clinical practice, despite limitations and possible inadequacies, therapeutic decisions are often based solely on variant pathogenicity (5–8). However, while pathology guidelines mark the importance of reporting specific tumor variant parameters with respect to variant description and interpretation (24, 25), these are not always taken into consideration in NGS reports. For instance, the Joint Consensus of the Association for Molecular Pathology, American Society of Clinical Oncology, and College of American Pathologists recommends that VAF and coverage, among other metrics, should be evaluated and clearly stated in the molecular testing report (25). These parameters seem to be critical for variant interpretation and clinical decision making, as shown in our study of clonal pathogenic variants.

VAF reflects the variant load in the examined sample, i.e., the rate of altered DNA molecules among all analyzed DNA molecules. VAF is a standard NGS metric that may help in interpreting the biological impact of the pathogenic variant in tumor tissues, provided that technical and sample issues are addressed (36, 38). FFPE tumor samples pose additional challenges due to DNA quality and to the presence of malignant and non-malignant cell DNA. The assessment of tumor burden or cellularity (TCC%), is performed by an experienced pathologist, manually or computationally, with a high concordance between the two approaches (39). In the present series, there were only 11 (2.2%) tumor samples without matched germline data and with cellularity below 30%, which is considered a safe tumor burden for variant interpretation (38). Pathogenic variants are considered clonal if present in all tumor cells (34). For example, in a tumor sample with 50% cellularity, a 25% VAF potentially indicates that the variant is present in all malignant cells or, if pathogenic in tumor suppressors, that gene function is lost in half of them. In the same tumor, a 5% VAF would potentially pertain to 10% or 20% of the tumor cells, respectively. This is an approximate approach for assuming the impact of a pathogenic variant in a given tumor, which, as shown here, seems of clinical relevance.

Current NGS technologies typically detect and report pathogenic variants that are present even at low rates. Low VAFs might result from normal cell contamination or tumor heterogeneity, being indicative of subclonal variants (40). Clonality has been previously shown to affect the prognostic and predictive role of the variant in patients with hematologic malignancies (41). Since clonality might interfere with the clinical utility of a variant, VAF needs to be considered for variant interpretation. However, VAFs are often not included in tissue genotyping reports. In line with previous studies (28), we observed that HRR variants were predominantly clonal in ovarian tumors, which is compatible with a driver role of HRR in the development of these cancers. Based on our study, clonal pathogenic variants represent only a subgroup of pathogenic HRR variants (21.6%), however they seem to indicate the proportion of patients who benefit the most from platinum treatment. Whether the remaining subclonal variants are predictive of benefit from platinum agents is worth further evaluating.

Driver co-existing mutations are often being identified in diverse tumor types. Whether co-occurrence of additional driver mutations interferes with the predictive role of specific mutations remains to be prospectively studied. Investigators have examined whether co-occurring mutations affect the predictive role of pathogenic variants in specific genes (42–44). In one study, patients with NSCLC with TP53/EGFR co-mutations had marginally shorter PFS when treated with EGFR inhibitors (42). Another study of patients with NSCLC who were treated with EGFR inhibitors also demonstrated that concurrent driver gene mutations were associated with poorer clinical outcomes (44). In patients with ovarian cancer, the presence of two concurrent driver mutations was also associated with significantly shorter time to relapse (29). Patients with co-mutations had more frequently platinum-refractory disease, compared to patients with one mutation. In line with previous findings, in our study patients with HRR/TP53 co-mutation had shorter OS compared to patients with HRR-only mutations. Even though patients with HRR-only clonal pathogenic variants represented a small proportion (7.7%) of patients with epithelial ovarian cancer, these were the ones to benefit the most and seem to have the best prognosis. Additional molecular alterations need to be taken into consideration when assessing the predictive role of selected mutations.

Our study had certain limitations, including the retrospective nature. Furthermore, debulking status, which is known to be strongly correlated with clinical outcomes in ovarian cancer, could not be included in the analysis due to missing data in a large proportion of our patients which would significantly limit the study’s sample size. For the same reason, we do not report on platinum sensitivity. Additionally, we do not address other molecular factors that might interfere with the predictive role of HRR pathogenic variants. The strengths of our study include the long follow up of our patients, large number of patients and the inclusion of diverse histological types of ovarian cancer.

## Conclusions

In conclusion, we demonstrated that variant clonality and co-occuring TP53 variants might affect the predictive value of HRR pathogenic variants for platinum agents, which probably applies to PARP inhibitors as well. Our findings emphasize the need to improve variant assessment and interpretation during routine tumor NGS testing, to enhance the predictive value of pathogenic variants in patients with EOC.

## Data Availability Statement

The data analyzed and presented in the study are publicly available. This data can be found here: https://files.hecog.gr/OVARIAN_Dataset.xlsx.

## Ethics Statement

The translational study protocol was approved by the Bioethic Committee of Aristotle University of Thessaloniki (AUTH) school of Medicine (Approval #79/10-06-2014) and by the Institutional Review Boards of “Papageorgiou” Hospital (Approval #193/15-01-2014) and “Thermi” Clinic (Approval #308/02-03-2016). The patients/participants provided their written informed consent to participate in this study.

## Author Contributions

Conceptualization: EF, VK, and GF. Formal analysis: G-AK, EG, and IT. Investigation: VK, KP, SC, KC, and FF. Resources: EF, ML, AP, DM, DP, GO, DA, AA, AV, GA, FF, and GF. Writing: EF, VK, G-AK, KP, FF, and GF. Supervision: EF, VK, and GF. Review and editing: all authors. All authors contributed to the article and approved the submitted version.

## Funding

This study was supported by Astra Zeneca and by an internal Hellenic Cooperative Oncology Group (HeCOG) research grant (TR_4G/20).

## Conflict of Interest

EF: Advisory Role: LEO Pharma. Speaker fees: Roche, Pfizer, AstraZeneca. Stock ownership: GENPREX INC, Deciphera Pharmaceuticals, Inc. Travel grant: Merck, Pfizer, and K.A.M Oncology/Hematology and DEMO. DP: Advisory Role: Roche, MSD, Astellas. Honoraria: Roche, MSD, Astellas. GA: Advisory Boards: Novartis, BMS, Roche Hellas, Astra Zeneca, Sanofi, Amgen, Genesis Pharma, Merck, Pfizer. GF: Advisory Board: Pfizer, Novartis and Roche. Honoraria: Astra Zeneca. Stock ownership: ARIAD, GENPREX, Daiichi Sankyo, RFL Holdings, FORMYCON. The funders had no role in the design of the study; in the collection, analyses, or interpretation of data; in the writing of the manuscript, or in the decision to publish the results.

The remaining authors declare that the research was conducted in the absence of any commercial or financial relationships that could be construed as a potential conflict of interest.

## Supplementary Material

Supplementary Figure 1
**(A)** Overall survival based on the presence of HRR and/or TP53 pathogenic variants in the entire cohort including patients with wild-type tumors or with pathogenic variants in other genes (excluding mucinous tumors). **(B)** Progression-free survival based on the presence of HRR and/or TP53 pathogenic variants in the entire cohort of first-line treated patients including those with wild-type tumors or with pathogenic variants in other genes (excluding mucinous tumors). **(C)** Overall survival based on the presence of clonal HRR and/or TP53 pathogenic variants in the entire cohort including patients with wild-type tumors or with pathogenic variants in other genes (excluding mucinous tumors). **(D)** Progression-free survival based on the presence of clonal HRR and/or TP53 pathogenic variants in the entire cohort of first-line treated patients including those with wild-type tumors or with pathogenic variants in other genes (excluding mucinous tumors).
