# Supplementary material for: Tumor Genotyping and Homologous Recombination Repair Gene Variants in Patients With Epithelial Ovarian Cancer: Is Pathogenic Enough?
Source: Front Oncol. 2021 Jun 1;11:683057. doi: 10.3389/fonc.2021.683057 (PMC8204021; doi:10.3389/fonc.2021.683057)
Supplement: Supplementary file 2 [file Table_1.docx]

**Supplemental Table 1.** Associations of the presence of (clonal) pathogenic variants with clinicopathological parameters.

|  | **Pathogenic variants** | | | | **Clonal pathogenic variants** | | | | **Position LOH** | | | **Clonal pathogenic mutations** | | |
| --- | --- | --- | --- | --- | --- | --- | --- | --- | --- | --- | --- | --- | --- | --- |
|  | **HRR only** | **TP53 only** | **Both** | ***p*** | **HRR only** | **TP53 only** | **Both** | ***p*** | **No** | **Yes** | ***p*** | **No** | **Yes** | ***p*** |
| **Age** | 50.5(26.5,76.7) | 59.3(28.4,83.5) | 55.7(27.5,83.9) | **<0.001** | 52.2(26.5,76.7) | 59.3(28.4,83.5) | 54.9(29.2,83.9) | **<0.001** | 57.6(21.7,82.9) | 58.0(26.5,83.9) | 0.33 | 57.9(21.7,82.9) | 57.7(26.5,83.9) | 0.73 |
| **Stage** |  |  |  | **0.024** |  |  |  | 0.38 |  |  | **0.034** |  |  | **<0.001** |
| I-II | 6(15.0) | 37(18.0) | 8(7.0) |  | 4(10.8) | 35(16.0) | 7(10.0) |  | 63(21.6) | 28(14.1) |  | 45(27.3) | 46(14.1) |  |
| III-IV | 34(85.0) | 168(82.0) | 107(93.0) |  | 33(89.2) | 184(84.0) | 63(90.0) |  | 228(78.4) | 171(85.9) |  | 120(72.7) | 280(85.9) |  |
| **PS** |  |  |  | 0.11 |  |  |  | 0.71 |  |  | 0.51 |  |  | 0.19 |
| 0 | 33(82.5) | 149(74.5) | 76(66.7) |  | 29(78.4) | 154(72.3) | 50(71.4) |  | 208(75.9) | 145(73.2) |  | 120(78.4) | 233(72.8) |  |
| 1-3 | 7(17.5) | 51(25.5) | 38(33.3) |  | 8(21.6) | 59(27.7) | 20(28.6) |  | 66(24.1) | 53(26.8) |  | 33(21.6) | 87(27.2) |  |
| **Histology** |  |  |  | 0.91 |  |  |  | 0.71 |  |  | **<0.001** |  |  | **<0.001** |
| HGSOC | 32(80.0) | 171(82.6) | 97(82.9) |  | 30(78.9) | 187(84.2) | 59(84.3) |  | 197(67.0) | 176(87.6) |  | 98(59.0) | 276(83.6) |  |
| Other | 8(20.0) | 36(17.4) | 20(17.1) |  | 8(21.1) | 35(15.8) | 11(15.7) |  | 97(33.0) | 25(12.4) |  | 68(41.0) | 54(16.4) |  |

**Supplemental Table 2.** Hazard ratios and 95% confidence intervals estimated by univariate Cox regression models with respect to OS and PFS for patients with available data upon exclusion of those with mucinous tumors.

|  | ***OS*** | | | ***PFS*** | | |
| --- | --- | --- | --- | --- | --- | --- |
|  | **Event/Total** | **HR (95% CI)** | **p-value** | **Event/Total** | **HR (95% CI)** | **p-value** |
| **Age** |  | 1.03 (1.02-1.04) | **<0.001** |  | 1.02 (1.01-1.03) | **<0.001** |
| **Family Other Cancer** |  |  |  |  |  |  |
| No | 184/283 | Reference | -- | 198/267 | Reference | -- |
| Yes | 104/157 | 0.99 (0.78-1.26) | 0.94 | 119/154 | 1.07 (0.85-1.34) | 0.57 |
| **Stage** |  |  |  |  |  |  |
| I-II | 29/81 | Reference | -- | 32/67 | Reference | -- |
| III-IV | 277/390 | 3.07 (2.09-4.51) | **<.001** | 305/383 | 2.85(1.97-4.10) | **<0.001** |
| **PS** |  |  |  |  |  |  |
| 0 | 197/339 | Reference | -- | 221/324 | Reference | -- |
| 1-3 | 104/116 | 2.41 (1.89-3.06) | **<.001** | 108/113 | 2.17 (1.72-2.74) | **<.001** |
| **Histology** |  |  |  |  |  |  |
| HGSOC | 257/375 | 1.58 (1.17-2.13) | **0.003** | 281/363 | 1.41 (1.06-1.87) | **0.017** |
| Other | 51/99 | Reference | -- | 58/89 | Reference | -- |
